# Complex Compression Fracture in the Thoracolumbar Junction: A Case Report

**DOI:** 10.7759/cureus.50836

**Published:** 2023-12-20

**Authors:** Kiril Ivanov, Mihail Kalnev, Petar-Preslav Petrov, Simeon Bashev, Plamen Penchev

**Affiliations:** 1 Faculty of Medicine, Medical University of Plovdiv, Plovdiv, BGR; 2 Neurological Surgery, University Multi-profile Hospital for Active Treatment (UMHAT) Saint George, Plovdiv, BGR; 3 Anatomy, Histology and Embryology, Medical University of Plovdiv, Plovdiv, BGR; 4 Faculty of Medicine, Medical University - Sofia, Sofia, BGR

**Keywords:** case report, vertebral stabilization, ct (computed tomography) imaging, thoracolumbar junction, vertebral compression fracture

## Abstract

Compression fractures in the thoracolumbar junction are one of the most frequent types of spine injuries. They can be the result of trauma or underlying conditions of the vertebrae. We present a case report of a 68-year-old patient with pain and loss of mobility in the lumbar spine after sustaining a trauma via falling from a significant height. Lumbar spondylography and a following CT scan revealed a complex compression fracture of L1 with degenerative osteoporotic changes of lumbar vertebrae and several pathologies of the lumbosacral junction. A surgical intervention was performed in the form of posterior transpedicular vertebral stabilization with titanium rods and screws. Postoperatively, relief from the lumbar region pain was reported. No neurological deficit was observed. The patient was mobilized, rehabilitated, and discharged from the hospital. This case report emphasizes the use of reliable imaging methods for the diagnosis of thoracolumbar compression fracture and highlights the reliability of surgical treatment of the condition via posterior transpedicular vertebral stabilization.

## Introduction

The thoracolumbar junction (T10-L2) is an area of great biomechanical stress and fractures in that area are the most frequently reported type of spinal fracture. The injuries in the junction, especially above and including the level of L1, often prove to have severe clinical manifestations, among which are pain, loss of function, and various deformities [1]. As such, adequate clinical examination and diagnosis are a necessity in treating this type of fracture. The use of reliable imaging methods such as CT scanning and MRI is highly encouraged for the purpose of diagnosing trauma of the lumbar spine, while radiography remains a usable first-line imaging modality for severe fractures that prove obvious to diagnose and lack suspicions for additional fractures or canal compromise [2]. Fractures in the thoracolumbar junction are observed mainly in females above the age of 50, commonly with coexisting pathologies such as osteoporosis, spondylosis, and spinal osteosclerosis [1,3]. Of these, nearly half are compression fractures, emphasizing the widespread occurrence of this particular injury [4]. In this case report, we present a case of a complex compression fracture of a vertebra in the aforementioned junction (L1) with various coexisting pathologies of the lumbar spine and assess the chosen surgical approach and the postoperative outcome. We aim to evaluate the reliability and effectiveness of the imaging modalities and the use of posterior dynamic vertebral stabilization systems for the treatment of severe thoracolumbar compression fractures in which additional fractures are also observed.

This article was previously presented as a meeting abstract at the XX International Medical Conference for Students and Young Doctors in Pleven, Bulgaria on 18 October 2023.

## Case presentation

**Figure 1:**
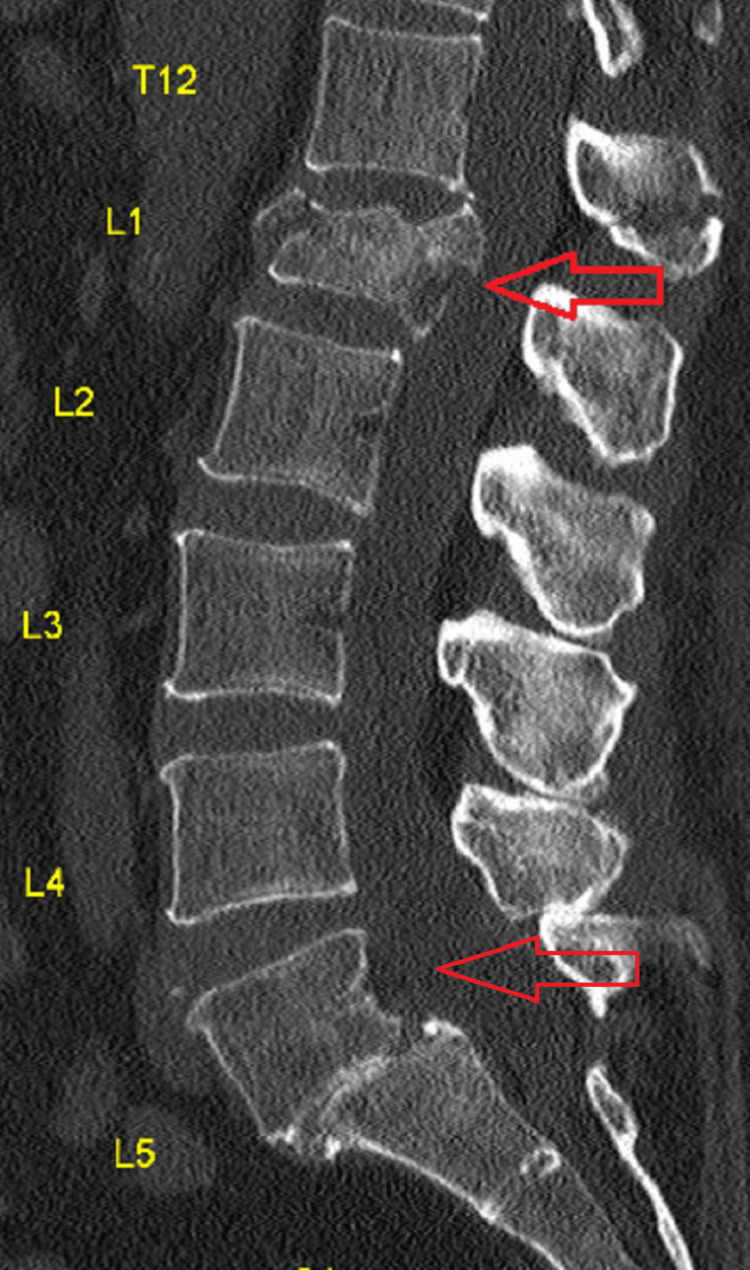
CT scan of the lumbosacral region from the sagittal plane, revealing evidence of a compression fracture of L1 alongside decreased intervertebral disc space and spondylolisthesis in the lumbosacral junction (L5/S1), with various degenerative changes of the lumbar vertebrae

**Figure 2:**
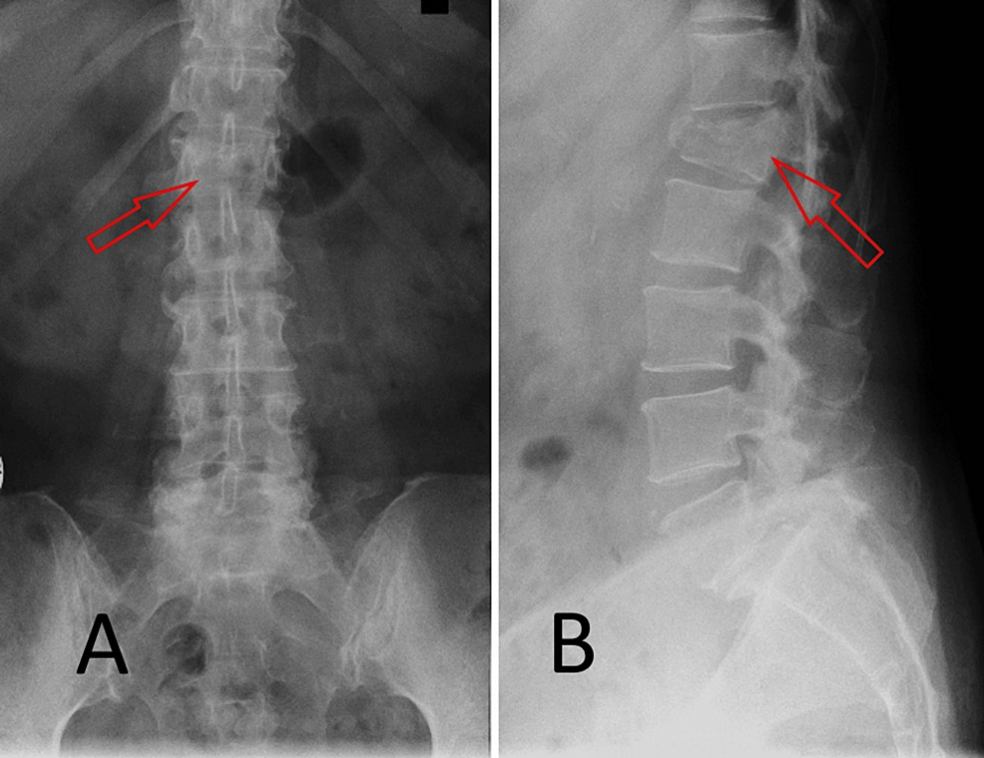
Spondylography of the the lumbar spine, showing the main compression fracture of L1. (A) Frontal plane. (B) Sagittal plane.

A 68-year-old female presented to the Neurosurgery Clinic with clinical manifestations of pain and restricted mobility in the lumbar region after sustaining trauma by falling from a height of one and a half meters (five feet) at home earlier the same day. The patient had several comorbidities of the cardiovascular system, such as unstable angina, a history of myocardial infarction, and hypertensive heart disease. The patient had no previous history of vertebral degeneration from osteoporosis or osteopenia. After a clinical examination that raised suspicions of a thoracolumbar fracture, radiography and a CT scan of the lumbar spine revealed evidence of a severe, mechanically unstable compression fracture of L1 in the thoracolumbar junction (Figures 1, 2A, 2B), with most of the vertebra's body and left lamina injured, alongside two additional fractures of the spinous process of T12 and the left transverse process of L2. Several coexisting pathologies were observed, among which а preexisting degenerative spondylolisthesis of the lumbosacral junction with severely decreased intervertebral space in L5/S1, subchondral osteosclerosis and degenerative changes of the spinal vertebrae due to the early stages of spondylosis. It was discovered that a fragment from the main fracture had compressed the structures of the spinal canal. The fracture was classified as L1 A4 as per the AO Spine Thoracolumbar Injury Classification System. 

**Figure 3:**
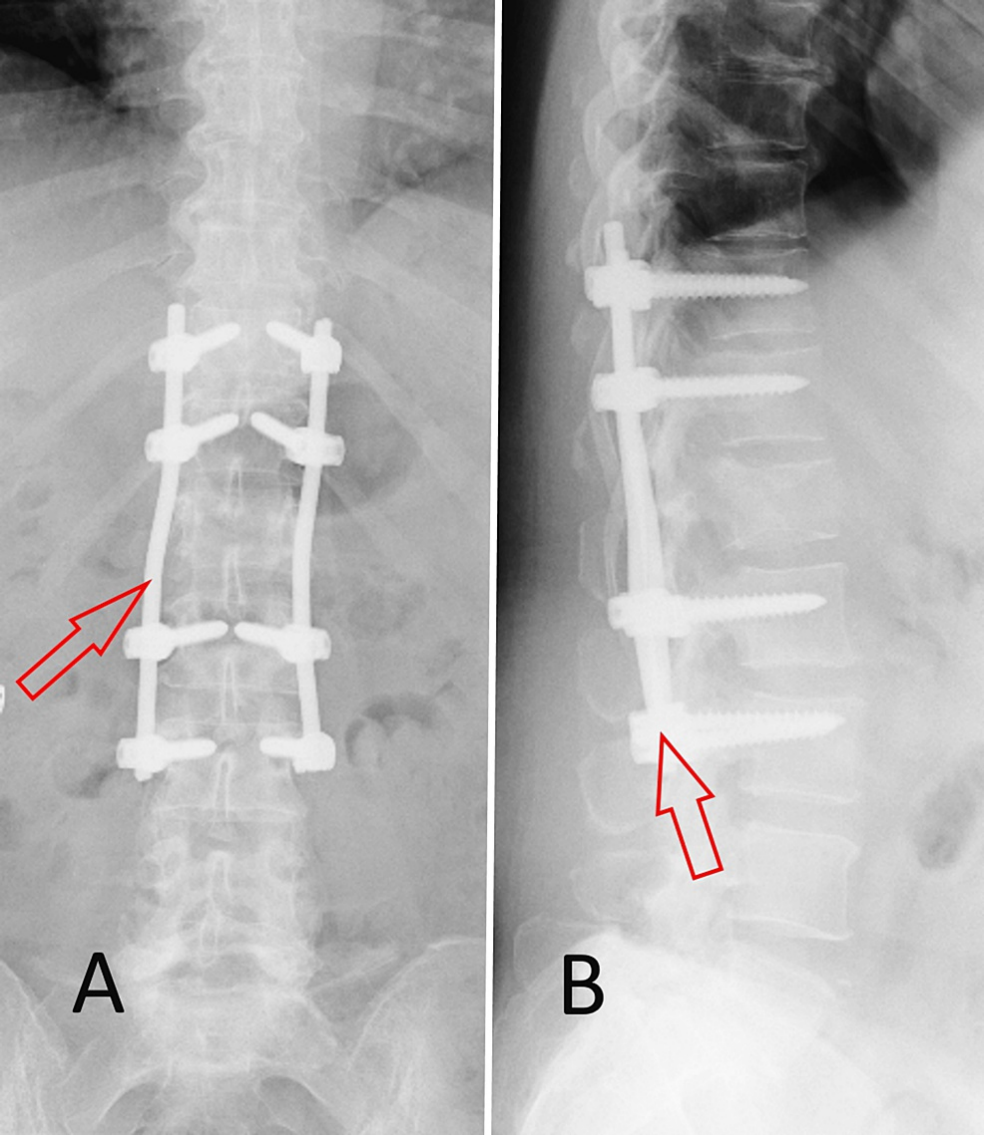
Postoperative spondylography of the thoracolumbar junction and the lumbar spine, demonstrating the completed posterior transpedicular stabilization system. (A) Frontal plane. (B) Sagittal plane

A surgical approach was chosen for the patient. Preoperatively, the patient was treated for her pain with tramadol 2x500 mg administered orally and acetaminophen 2x500 mg orally, alongside dexamethasone i.v. Under general anesthesia, posterior transpedicular stabilization was performed by implanting titanium rods and screws into the patient’s spine on the level of T11,12-L2,3 (Figure 3). A definitive vertebral stabilization was achieved. Postoperatively, the patient reported relief in lower back pain, alongside the emergence of pain in the left inguinal crease upon movement which later subsided. No neurological deficit was observed. The patient was successfully verticalized and mobilized. An individual rehabilitation program was appointed consisting of physical therapy including abdominal isometric exercises, diaphragmatic breathing and quad exercises (long and short arc). After five days, the patient was discharged from the hospital. The two follow-up examinations (on the 15th and 30th day after the surgical intervention) showed satisfactory overall recovery of the patient with regard to pain, mobility, and function without new clinical manifestations.

On the 15th-day follow-up examination, the patient reported the use of over-the-counter painkillers, namely metamizole 2x500 mg orally daily, alongside moderate pain in her lumbar region when moving and a recurrence of her inguinal crease pain which subsided completely around the 12th day. Her mobility had increased to a satisfactory level, with her being able to tolerate up to 6-8 minutes of mild cardiovascular activity such as continuous walking while keeping a neutral position of the spine, and without any significant worsening of her lumbar pain to be reported. Her postoperative wound showed some redness and minimal swelling without any signs of an ongoing bacterial infection; the stitches of the wound were taken off. The need for the patient to wear a spinal brace was reinforced. On the 30th-day follow-up examination, the patient reported further subsiding of any pain to the point when it was classified as mild while using metamizole 1x500 mg (orally) infrequently for pain relief. Her tolerance for mobility had increased up to 10 minutes of mild cardiovascular activity while keeping a neutral spine position. No complication of any comorbidities was reported by the cardiologist consulting her. A choice was made for the patient to continue wearing a brace for another two weeks. A further rehabilitation plan for the patient was drafted, including the time for up to 12 weeks postoperatively, with the goals of lumbar muscle strengthening and stability routines, including exercises such as abdominal bracing and isometrics, neural mobilization exercises, functional training, and a supine position exercise protocol, including supine marches, heel slides, and hip abductions. The walking exercise was emphasized. There were no signs of a neurological deficit reported or any irregularities of the bone healing process, despite the complexity of the main fracture, the canal compromise, and the additional process fractures.

## Discussion

Due to anatomical factors, namely the transition between the dynamic, mobile lumbar spine and the more static thoracic spine, the thoracolumbar junction (T10-L2) proves to be the area with the most numerous spinal fractures, as much as 90% of all reported injuries of this kind [1,3]. Of these fractures, nearly half (47.81%) are composed of compression fractures [4]. 40% to 80% of thoracolumbar injuries occur in settings of high energy (collisions, falls, etc.) and often lead to neurological deficits and additional trauma [1]. The severity and complexity of such injuries, combined with their relative frequency of occurrence, require adequate clinical examination and the use of reliable imaging modalities for adequate diagnosis.

Misdiagnosis and underdiagnosis of vertebral compression fractures (VCFs) remain an acute problem, as approximately two-thirds of them are not properly diagnosed and remain untreated [3]. Therefore, the choice of a reliable imaging method for diagnosis is highly emphasized. Spondylography remains a popular choice, although it is cited by multiple studies as unreliable by itself and prone to failure in vertebral fracture assessment, with a misdiagnosis rate of up to 50% [3,5]. It remains a viable first-line imaging modality for severe fractures that are clearly seen, especially when there are no suspicions of canal compromise or additional fractures, given its wider availability in hospitals worldwide. CT scanning, on the other hand, yields superior image quality and higher diagnostic accuracy which gives more anatomical information about the lumbar spine in particular [6]. MRI provides superior-quality intramedullary pathology detection and proves to be a useful method of evaluation of spine trauma in general, although it lacks the capacity for the depiction of bony details of CT scanning [7]. The shorter duration of a CT scan should be taken into consideration when choosing an imaging modality, and it is recommended as a primary screening method when there are contraindications for the use of MRI.

Coexisting conditions should be taken into account when planning the treatment for thoracolumbar injuries. The most common of these conditions is osteoporosis, which is reported as a primary cause of VCFs in elder patients, females in particular [3,8]. Dynamic vertebral stabilization via metal rods is reported to be a satisfactory and reliable method of treatment for VCFs, even when there are severe degenerative changes of the vertebrae, as it offers a lower amount of shielding and a motion of the spine closer to normal than the rigid alternatives [8]. The posterior approach to vertebral stabilization of thoracolumbar fractures in particular provides more satisfactory functional outcomes for the patients in the long-term (in the case of the study - a two-year postoperative follow-up) in comparison with anterior or combined posteroanterior systems [9].

Despite the reliability and efficacy dynamic vertebral stabilization is proved to have, it is prone to cause adjacent segment degeneration (ADS) in the lumbar spine, with a rate between 7% and 28% in the studies with a follow-up period higher than 60 months, which may require revision surgery (in up to 12.8% of cases); other postoperative complications associated with this treatment modality include device breakages, either of the screw or the rod (2,3% of reported cases), or screw loosening (with an up to 17% occurrence, of which 12,9% require revision surgery), a complication which in itself can prove asymptomatic but cause risk for infections [10]. These clinical outcomes and complications demonstrate that dynamic vertebral stabilization can lead to similar biomechanical issues as spinal fusion surgery, all the while retaining a higher level of spine mobility for the patients.

Alternative treatment modalities for VCFs include conservative treatment, vertebroplasties, and kyphoplasty. Conservative treatment is not well-defined by clinicians and can include physical therapy, manual therapy, and opioid pain management; there is a lack of information on this treatment modality in clinical literature in the case of traumatic VCFs of the thoracolumbar junction in particular and further research is desirable and needed in order to draw a conclusion on the matter [11]. Vertebroplasty, while offering a minimally invasive solution for unstable osteoporotic VCFs in particular, is questioned in its efficacy by the medical community, as at least one study concludes that it yields no significant benefits over a sham (placebo) surgery, resulting in little to no improvement in the patient's mobility, quality of life and pain while in some cases having severe adverse effects and clinical complications, among which cardiopulmonary disease (highly relevant for our patient), ADS, retropulsion of bone into the spinal canal, severe rigid collapse and cement leakage [1,12]. Kyphoplasty has similar risks to vertebroplasty, although it is generally deemed the safer procedure, with a symptomatic complication rate of up to 3.9% and no significant pain or function improvements for most patients [1]. However, one study emphasizes the benefits of kyphoplasty combined with posterior dynamic stabilization, as the first procedure offers augmentation of the vertebral columns and restoration of vertebral height, while the stabilization device allows better load sharing, stabilization of the injured segment and better mobility for adjacent segments [8].

When it comes to our patient's long-term clinical outcomes, they proved satisfactory, although several factors are to be taken into consideration for the recovery and rehabilitation of elderly patients with unstable thoracolumbar VCFs. The patient's cardiovascular comorbidities did not lead to complications, in accordance with the clinical literature, in which a connection between posterior dynamic vertebral stabilization and cardiopulmonary complications and pathologies is not observed. The comorbidities were, however, taken into consideration when drafting the patient's rehabilitation routine, alongside the more significant factors in this case, namely the coexisting pathologies of the patient's lumbar spine and mainly the degeneration of the vertebrae caused by spondylosis. The degenerative spondylolisthesis of the patient was also accounted for but did not lead to significant changes in the exercise routine, as both flexion and stabilization exercises yield similar recovery results in patients with this type of spondylolisthesis [13]. The walking exercise was incorporated into the patient's rehabilitation routine after she was discharged from the hospital, as when combined with lumbar stabilization circuits it significantly improves back muscle endurance and core stability [14], something important given the patient's copathologies of the lumbar spine and the long-lasting back pain reported post-operatively after thoracolumbar stabilization surgery. Given the spondylosis of the patient, a longer therapy window was planned, and exercising in the supine position was emphasized. Her inguinal pain on the left subsided completely before the first follow-up examination and was ruled as idiopathic. Overall, the patient's recovery was conducted according to guidelines and proved satisfactory, given her comorbidities, the nature of the main fracture, and the two additional fractures.

Based on the information presented so far, it is our recommendation that complex compression fractures in the thoracolumbar junction should be treated surgically with dynamic posterior stabilization systems to achieve the best possible postoperative results in the aspects of movement and functionality of the patient’s spine and lower back.

## Conclusions

VCFs in the thoracolumbar junction prove to be a common problem worldwide that remains unresolved at least in part due to poor imaging methods causing underdiagnosis, misdiagnosis, and improper treatment, both surgically and nonsurgically. We recommend that radiography by itself, while useful in the context of high-energy trauma and severe fractures, is an unreliable imaging method and should be used alongside CT and/or MRI. While surgical treatment by rigid spinal stabilization is a viable and effective option, dynamic systems prove to have superior results for the patient’s mobility while risking similar adverse effects. Vertebral stabilization from the posterior of the spine is the recommended surgical approach for the treatment of thoracolumbar injuries as opposed to anterior or combined interventions, as it yields better subjective and functional outcomes when performed.

In the case presented by us, an elderly female patient with a traumatic, mechanically unstable VCF in the thoracolumbar junction with several copathologies of the lumbar spine and two additional process fractures managed to make a satisfactory recovery via posterior dynamic vertebral stabilization and regained an adequate amount of mobility and function during the first month postoperatively with the aid of an individualized rehabilitation program and exercise routine, which managed to negate the biomechanical and anatomical disadvantages caused by her age, lumbar spondylosis, and degenerative spondylolisthesis.
